# Congenital communicating bronchopulmonary foregut malformation including ectopic pancreatic tissue in an infant

**DOI:** 10.1186/s40792-021-01211-w

**Published:** 2021-05-24

**Authors:** Noboru Oyachi, Fuminori Numano, Keiichi Koizumi, Tamao Shinohara, Hirochika Matsubara

**Affiliations:** 1 Department of Pediatric Surgery, Yamanashi Prefectural Central Hospital, 1-1-1 Kofu, Yamanashi, 409-8506 Japan; 2 Department of Pediatrics, Yamanashi Prefectural Central Hospital, Kofu, Japan; 3 Department of Thoracic Surgery, University of Yamanashi, Chuo, Japan

**Keywords:** Bronchopulmonary foregut malformation, Ectopic pancreatic tissue, Esophagus, Pulmonary sequestration, Infant

## Abstract

**Background:**

Several reports have documented that the pulmonary sequestration is in communication with the gastrointestinal tract and the concept of bronchopulmonary foregut malformation (BPFM) has become more widespread. However, there are few reports of the sequestration associated with the pancreas derived from the foregut. We describe the history and pathophysiology of BPFM including pancreatic tissue in a male infant with respiratory distress.

**Case presentation:**

A male patient was born at 38 weeks of gestation and weighed 2752 g at birth. He developed pneumonia and was hospitalized at 3 months of age. Chest radiographs and CT scans led to the diagnosis of a lung abscess in the left lower intralobar pulmonary sequestration with aberrant arteries from the abdominal cavity. At 4 months of age, when the abscess had resolved, left lower lobectomy and the resection of the intralobar sequestration were performed. The pulmonary sequestration was conjoined with the esophagus. A fistula was found between the lower esophageal wall and the pulmonary sequestration. An additional small segment of the esophageal wall was excised. Histologically, the mediastinal surface of the sequestration tissue contained pancreatic tissue. Furthermore, esophageal and gastric tissue, cartilage tissue, and ciliated epithelium were confirmed. A definitive diagnosis of BPFM was made.

**Conclusions:**

We postulated the rare case of a communicating BPFM with intrapulmonary sequestration on one end and the esophagus on the other forming a mass lesion, which included ectopic pancreatic tissue in a male infant.

## Background

Bronchopulmonary foregut malformation (BPFM) refers to a very rare spectrum of congenital defects that are due to abnormal differentiation of the respiratory and upper gastrointestinal tracts during early embryonic development [1].

Common embryologic pathogenesis causes a wide range of malformations in BPFM, including pulmonary sequestration with partial or complete communication to esophagus or stomach, esophageal or gastric diverticulum, and esophageal or bronchogenic duplication cysts [2].

Although several reports have documented that the pulmonary sequestration is in communication with the gastrointestinal tract, there are few reports of the sequestration associated with the ectopic pancreas derived from the foregut.

We describe the history and pathophysiology of BPFM including ectopic pancreatic tissue in a male infant with respiratory distress.

## Case presentation

**Fig. 1:**
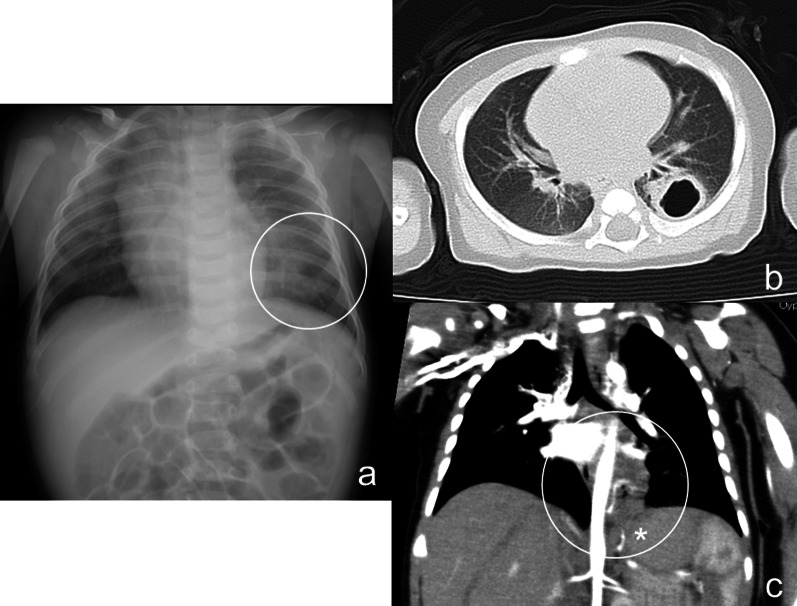
The chest radiograph showed a ring-shaped infiltration 2 cm in diameter in the lower left lung field (**a**). Contrast-enhanced computed tomography (**b**, **c**). CT scans led to the diagnosis of a lung abscess in the left lower intralobar pulmonary sequestration forming a tumor-like lesion with aberrant arteries (asterisk) from the abdominal cavity

**Fig. 2:**
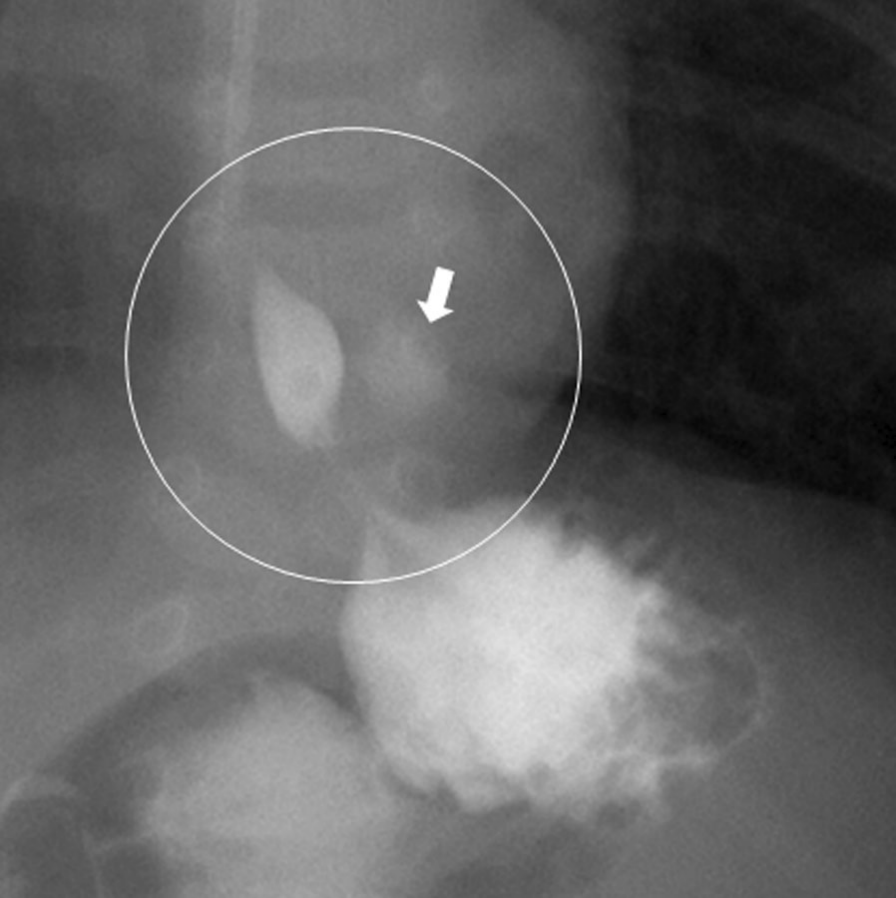
Esophagogastric cotrast study. The esophagogastric cotrast study revealed a faint contrast structure (arrow) just above the left diaphragm and in contact with the esophagus

The male patient was born at 38 weeks gestation and weighed 2752 g at birth. The patient had a history of bloody vomiting from 2 months of age and was initially diagnosed with gastroesophageal reflux disease. The patient developed pneumonia and was hospitalized at 3 months of age. A chest radiograph showed a ring-shaped infiltration 2 cm in diameter in the lower left lung field and contrast-enhanced computerized tomography (CT) scans demonstrated a lung abscess in the left lower intralobar pulmonary sequestration forming a tumor-like lesion with aberrant arteries from the celiac trunk (Fig. 1). Review of our past esopahgogastric contrast study of the patient revealed a faint contrast structure in contact with the esophagus just above the diaphragm (Fig. 2). We suspected that this contrasted structure was the pulmonary sequestration in BPFM. First, we planned to treat with antibiotics and then surgically remove the lesion after the inflammation had subsided, because the patient’s respiratory condition was stable.

**Fig. 3:**
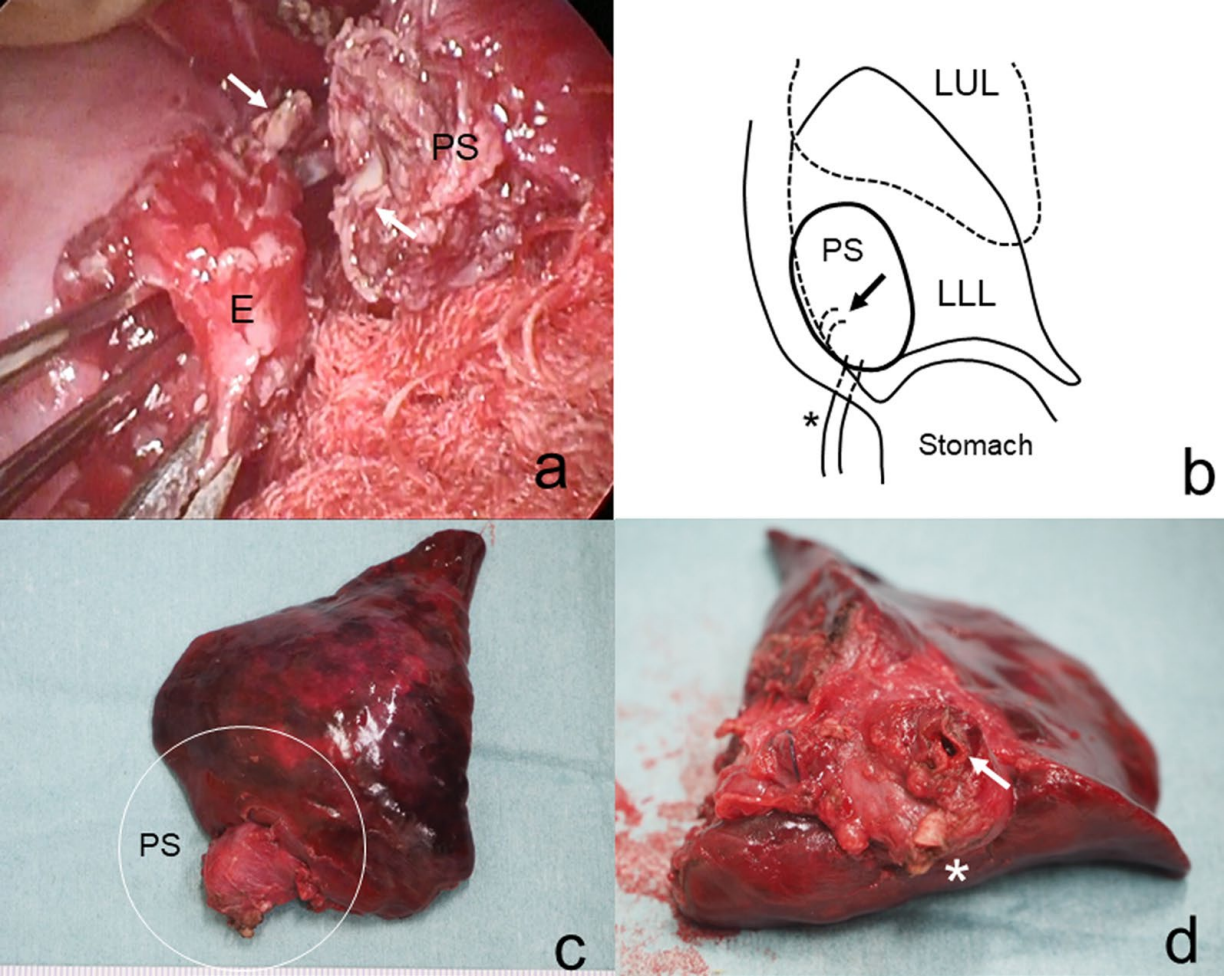
Operative findings (**a**), schema (**b**) and resected specimen (**c**, **d**). Left lower lobectomy and resection of the intralobar pulmonary sequestration were performed. The pulmonary sequestration and surrounding tissues were strongly adherent and were conjoined with the esophagus. A fistula was found between the lower esophageal wall and the pulmonary sequestration. A small segment of the esophageal wall was excised. (PS: pulmonary sequestration, E: esophagus, arrow: fistula, asterisk: aberrant artery)

At 4 months of age, when the abscess had resolved with antibiotics, left lower lobectomy including intralobar sequestration was performed. The pulmonary sequestration and surrounding tissues in the mediastinum were strongly adherent and became a mass lesion. Moreover, the mediastinal surface of the sequestration merged into the esophagus. Left lower lobectomy including the mass lesion was considered unavoidable because no boundaries were defined between them. The lower lobe of the left lung, including intralobar isolation (S10) with a 1 cm length of the esophageal wall, was resected. A fistula of 3 mm diameter was found between the lower esophageal wall and the pulmonary sequestration (Fig. 3).

**Fig. 4:**
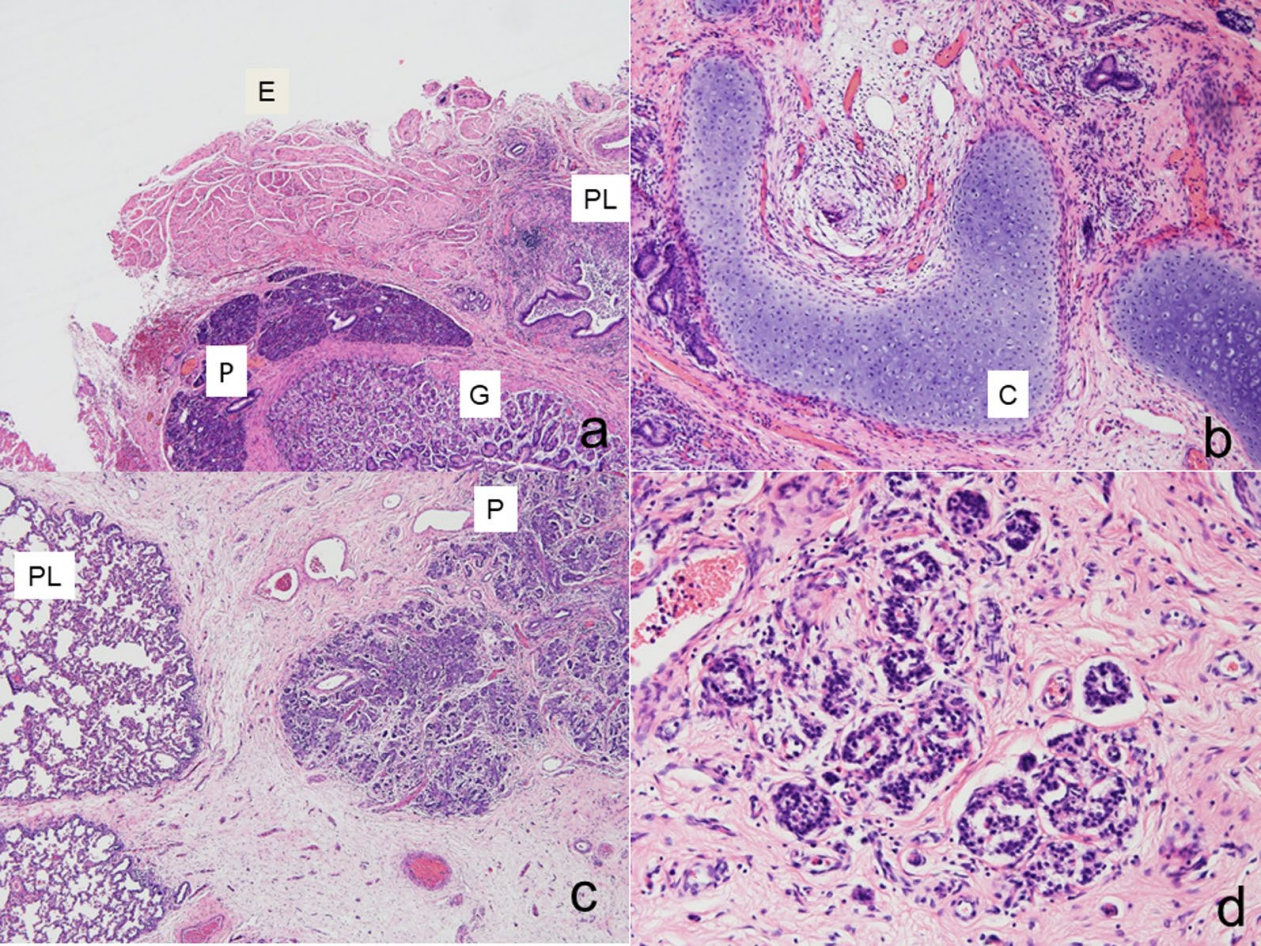
Pathological findings. Histologically, the mediastinal surface of the sequestration was mostly composed of pancreatic tissue (P). Esophageal (E) and gastric (G) tissue, cartilage tissue (C) and ciliated epithelium were confirmed in pulmonary tissue (PL) (**a**: H&E × 40, **b**: H&E × 100, **c**: H&E × 40). All pancreatic tissue components including islets of Langerhans were identified (**d**: H&E × 200)

Histologically, a mass lesions attached to the esophagus and the intrapulmonary sequestration contained ectopic pancreatic tissue, which were scattered within the fibrovascular connective tissue and the morphology was similar to H&E stained pacreatic tissue reported by Ballouhey [3] including islets of Langerhans, acinus, and ductal tissue. Gastric mucosa, tracheal cartilage, and ciliated columnar epithelium were also randomly distributed in the specimens (Fig. 4). A definitive diagnosis of BPFM was made.

After the operation, the patient is doing well and has no respiratory or swallowing problems.

## Discussion

Pulmonary sequestration which communicates with the esophagus has previously been reported as a form of BPFM, and various pathological conditions associated with BPFM have been reported, such as communication with the esophagus and lung sequestration in the abdominal cavity [1, 2, 4]. This case demonstrates a unique example of a BPFM which forms a tumor-like or mass lesion, including the esophageal wall and intrapulmonary sequestration containing ectopic pancreatic tissue derived from the foregut.

In this case, surgery was performed after the formation of an abscess. The esophagus and sequestrated lungs fused fibrously, forming a mass lesion containing a fistula and this condition has been reported in a small number of patients with BPFM [5, 6]. The resected specimen in our case contained pancreatic tissue consisting of exocrine and endocrine tissue. There have been four previous case reports of pancreatic tissue within BPFM [3, 7–9]. There have also been case reports stating that the ectopic pancreas causes peripheral inflammation [10]. We also speculated that the chronic inflammatory reaction caused by the ectopic pancreas may have formed tumor-like structures. However, blood data did not show an increase in pancreatic enzymes.

Bloody vomiting in infancy is a typical sign of gastroesophageal reflux disease. Our case was initially diagnosed with gastroesophageal reflux, but eventually was accompanied by hemoptysis. Hemoptysis is rare in infancy and BPFM is one of the differential diagnoses. Therefore, CT scans and esophagography are required as imaging studies, leading to an early diagnosis. Attention should be paid to the structure of the esophageal wall when obtaining an upper gastrointestinal series.

## Conclusions

We described here the rare case of a communicating BPFM with intrapulmonary sequestration on one end and the esophagus on the other, including ectopic pancreatic tissue in a male infant. Although the concept of BPFM has become more widespread, the effects of ectopic pancreatic tissue in BPFM are unclear. The accumulation and analysis of additional cases is needed.

## Data Availability

The dataset supporting the conclusion of this article is included within the article.

## Abbreviation

BPFM: Bronchopulmonary foregut malformation
